# Oromucosal Administration of Interferon to Humans

**DOI:** 10.3390/ph3020323

**Published:** 2010-01-28

**Authors:** Manfred W. Beilharz, Martin J. Cummins, Alayne L. Bennett, Joseph M. Cummins

**Affiliations:** 1 School of Biomedical, Biomolecular and Chemical Sciences, University of Western Australia, 35 Stirling Highway, Crawley WA 6009, Australia; E-Mail: bennetta@cyllene.uwa.edu.au (A.L.B.); 2 Amarillo Biosciences, Inc., 4134 Business Park Drive, Amarillo, TX 79110, USA; E-Mails: mcummins@amarbio.com (M.J.C.); jcummins@amarbio.com (J.M.C.)

**Keywords:** interferon, oromucosal delivery, influenza, treatment, viral diseases

## Abstract

The prevailing dogma is that, to be systemically effective, interferon-alpha (IFNα) must be administered in sufficiently high doses to yield functional blood concentrations. Such an approach to IFNα therapy has proven effective in some instances, but high-dose parenteral IFNα therapy has the disadvantage of causing significant adverse events. Mounting evidence suggests that IFNα delivered into the oral cavity in low doses interacts with the oral mucosa in a unique manner to induce systemic host defense mechanisms without IFNα actually entering the circulation, thus reducing the potential for toxic side effects. A better understanding of the applications and potential benefits of this treatment modality are under active investigation. This paper provides a review of the relevant literature on the clinical use of the oromucosal route of administration of interferon, with an emphasis on the treatment of influenza.

## 1. Introduction

Injections of interferon alpha (IFNα) by intravenous, subcutaneous, intramuscular, and intraperitoneal routes of administration have all been used to treat numerous diseases [1,2]. Topical and localized IFNα administration (skin ointments, eye drops, intranasal sprays and intralesional injection) have been used against localized diseases (e.g., colds, influenza, ocular herpes, warts) [2,3,4]. The prevailing dogma is that, to be systemically effective, IFNα must be administered in sufficiently high doses to yield functional blood concentrations. Such an approach to IFNα therapy has proven effective in some instances, but high-dose parenteral IFNα therapy has the disadvantage of causing significant adverse events [5].

Several early pharmacokinetic studies of IFNα given by various routes to animals reported that "orally" administered IFNα was not detectable in the systemic circulation [6,7,8,9,10]. However, the investigators of these studies did not deliver the IFNα by the oral mucosal route. Instead, they by-passed the oral cavity by administering the IFNα by gavage directly into the stomach. This by-passed involvement of any potential immunologic mechanism(s) that would be activated by contact of IFNα with the oral mucosa. It has been reported that IFNα administered into the oral cavity protected cattle from virulent virus challenge, while IFNα given directly into the rumen did not [11].

The presence of detectable IFNα in the blood may not be required or even desired for IFNα administered by the oral mucosal route to activate systemic host protective mechanisms. Low doses of IFNα, given in the oral cavity to promote mucosal contact, do not produce measurable blood levels of IFNα.

IFNα delivered into the oral cavity may interact with the oral mucosa in some unique manner to induce systemic host defense mechanisms without IFNα actually entering the circulation. Since the oral mucosa is a primary interface between infectious agents and the host's defense system, oral mucosal cells are capable of producing IFNα in response to infectious agents and are able to respond to the IFNα produced [12]. Because of the frequent exposure of oral mucosal cells to potential pathogens, IFNα may interact often or uniquely with oral mucosal cells [13]. Therefore, the oral mucosal route of administration for IFNα may offer potential therapeutic benefits by inducing systemic host protective mechanisms. Numerous IFN-stimulated genes (ISG) are activated by oral IFNα in mice [14,15], cattle [16], hogs [17], fish [18], and man [19].

The first report of the efficacy and safety of oral IFNα was published in 1972 when it was observed that suckling mice receiving IFNα in the milk were protected against fatal viral challenge [20]. This publication motivated the oral use of bovine IFNα in two women with advanced melanoma [21]. The surprising recovery of these two women led to oral IFNα studies in cats, dogs, horses, hogs, cattle, poultry and rodents [22].

The localization and fate of various IFNs following parenteral administration has been reported. Radiolabeled IFNα given intravenously (iv) to humans accumulated in the saliva, oral cavity, nose and paranasal sinuses [23,24]. The biological basis for this localization is not clearly understood but it may represent part of an innate immune response to infections [25,26,27,28]. This innate immune response involves the nasal production of Type I IFNs (α and β) followed by their binding to oromucosal surfaces, which in turn activates both local and systemic responses. Based on this innate defense mechanism, IFNα and IFNβ have been administered orally to humans in various contexts and these studies are reviewed in this article. 

Some limited studies have also looked at the effects of oromucosal administration of IFN on cells of the oral cavity. Human IFNα (HuIFNα) upregulates expression of aquaporin-5 in human parotid glands *in vitro* [29]. When volunteers received oromucosal doses of HuIFNα, extracted buccal epithelial cells exhibited IFN stimulated gene-15 (ISG-15) transcription and production *in vitro* [30]. ISG-15 is a 15-kDa protein that is transcriptionally regulated by type I interferons [31,32]. HuIFNα increases HLA-DR expression in these same cells *in vitro* [33]. Since ISG-15 is known to induce IFNγ, oromucosal administration of IFNα may lead to enhanced IFNγ production and increased natural killer (NK) cell activity [30]. It is notable that either inhibition or promotion of IFNγ activity by IFNα/β has been observed, depending on the experimental circumstances [34]. 

Twenty volunteers were given placebo or HuIFNα oromucosally at 10^3^, 10^5^ or 10^7^ IU once daily for 7 days. The 1 mL oral doses were held in the mouth for 3 minutes before swallowing. Changes in lymphocyte counts, β-2 microglobulin concentrations and NK cell-activity led the investigators to conclude that the lower doses (10^3^ IU) were immunostimulating and the higher doses (10^7^ IU) were immunosuppressive [35].

In another study, 20 volunteers were given placebo or HuIFNα (150 or 450 IU) twice daily or three times daily (tid) for 1 or 5 days. Placebo or HuIFNα solutions were held in the mouth for 2 minutes before swallowing. Patients given HuIFNα, but not placebo, had increases in percentages and/or absolute values of CD3+, CD4+, CD8+, CD25+ and/or HLA-DR+ lymphocytes after therapy [36].

Five healthy human volunteers were given oral administration in a tablet with 125 IU of leukocyte IFN. Each healthy volunteer sucked on one tablet until it dissolved in the mouth and was absorbed into the oral mucosa. Surprisingly, the article reported that 2'.5'-oligoadenylate synthetase (2-5OAS) activity in whole blood of subjects given the IFN tablets increased after 5 hours and reached 4-8000 pM/dl. The increase in 2-5OAS activity after oral administration of 125 IU IFN was generally greater than the increase in 2-5OAS activity in four subjects given intramuscular injections of millions of IU of IFN. Thus, tiny oral doses of IFN resulted in more 2-5OAS activity than 80,000 times more injected IFN [37].

## 2. Oromucosal IFN Therapy in Influenza

In a study by Soloviev in 1967, natural (leukocyte) HuIFNα was given in low doses intranasally for three consecutive days to 374 subjects "at the height" of an influenza outbreak [38]. IFNα-treated subjects had less severe illness than 382 subjects given placebo. When IFNα or placebo was given to 637 subjects before the influenza outbreak, subjects given IFNα had less illness than the 317 subjects given placebo. Soloviev reported that the IFNα treatment was free of adverse events and proposed that IFNα “will be given proper place in the arsenal of means for fighting virus infections.”

In 1969, it was reported that approximately 14,000 people participated in controlled studies of placebo versus low-dose IFNα treatment during a natural outbreak of Hong Kong influenza [39]. Placebo or IFNα was dripped into the nose daily for five days starting about the time of the first reported influenza cases. The low-dose IFNα treatment significantly (p < 0.01) reduced the number of influenza cases in children and adults. 

In September 1971, a group of U.S. scientists visited the Soviet Union and reported that there was advanced clinical work on the use of exogenous IFNα [40]. Furthermore, the U.S. delegation reported that HuIFNα was available through pharmacies in the Moscow area for use as a nasal spray against influenza. 

Another group of U.S. scientists arrived in Moscow on January 20, 1973, during the waning days of an extensive influenza epidemic [41]. During the peak of the epidemic, the number of influenza cases reported in Moscow reached 90,000 per day. It was reported that, for three years, several Soviet medical centers observed that HuIFNα was effective in the prophylaxis of influenza. When low-dose HuIFNα treatment, given by nasal spray tid for three days and then once daily for two days, was started in a factory or school immediately after the first case of influenza, an approximately 60% decrease in influenza symptoms was reported in IFNα-treated patients, without adverse events.

To achieve therapeutic effects, HuIFNα was given both by aerosol and orally. At the first sign of influenza illness, HuIFNα was given by the oral and nasal aerosol routes. This was repeated in two hours if the patient's symptoms were severe and was always followed by intranasal administration of HuIFNα twice daily for three days. Clinicians reported that the HuIFNα treatment caused symptoms to disappear more quickly; fever and headache were reported to clear almost immediately [41]. 

In 1976, Arnaoudova reported from Bulgaria on the therapeutic and prophylactic benefit of low-dose HuIFNα given five times a day for three days (therapeutic) or given three times a day for three days repeated twice at 10-day intervals (prophylactic) [42]. No allergic or adverse events were observed in any of 868 children, including newborns and premature babies given IFNα during a natural outbreak of influenza A (Port Chalmers variant). The author reported that IFNα therapy reduced the severity and duration of disease, especially if started on the first day of illness. The author also reported that IFNα was effective in preventing influenza [42].

Intranasal drops of HuIFNα (5,000 IU) given daily for four months reduced the frequency and severity of disease due to influenza A (H_3_N_2_ and H_1_N_1_) and parainfluenza virus [43]. Data were collected on 83 volunteers in the study. Fever occurred in six of 39 volunteers given IFNα and in 15 of 44 volunteers given placebo (p < 0.02). Subjective symptoms such as headache, cough, fatigue, anorexia, myalgia, *etc*. occurred in only 34% of volunteers given IFNα compared to 67% of volunteers given placebo (p < 0.01).

In 1982, HuIFNα (5,000 IU/dose) or placebo was dripped into the nostrils of 27 children twice daily for 60 days [44]. The children lived in an orphanage where natural outbreaks of influenza A and influenza B occurred during the treatment period. The HuIFNα did not prevent illness but did significantly reduce the duration of fever and the mean peak fever. Clinical manifestations of influenza were milder in children given IFNα, compared to placebo. Adverse events due to HuIFNα therapy were not observed.

During influenza epidemics in 1983, 1984 and 1985, 140 children were treated with a spray of natural HuIFNα, 700–1,600 IU/treatment, administered into the nose and mouth twice daily for 3–4 days [45]. The 53 control children were given traditional Chinese herbs. Children given HuIFNα had a significantly (p < 0.01) faster normalization of temperature at 24, 36 and 48 hours after the first treatment. The clinicians reported that pharyngitis and lymphadenosis of the posterior pharynx improved when fever subsided.

Subsequent studies that employed significantly higher doses of HuIFNα, usually 1,000–10,000 times more per day, did not report any benefit of oral HuIFNα therapy [46,47,48,49,50].

Recent animal studies reported that oral or intranasal IFNα was safe and effective against influenza virus in mice [51,52,53,54], guinea pigs [55] and ferrets [56]. Oral delivery of murine IFNα at 100 IU (p < 0.05) or 1000 IU (p < 0,015) to C57BL/6D mice reduced viral replication after intranasal inoculatioin of mouse-adapted human influenza virus (H1N1). Moreover, the oral dose of 100 IU of murine IFNα given once daily for 7 days after challenge protected mice from a lethal challenge of virus [51].

A single intranasal dose of 10,000 IU of hybrid HuIFNαB/D protected all 8 treated BALB/c mice that were Mx1^+/+^ when HuIFNαB/D was given 8 hours before challenge with 1000 LD_50_ of strain VN1203 of influenza A virus. The HuIFNαB/D did not protect mice that were Mx1^-/-^ [52]. In another study of Mx1^+/+^ mice, it was reported that a single intranasal dose of 500,000 IU of HuIFNαB/D, given 10 hours before the virus challenge, protected mice against a lethal challenge with 100 LD_50_ of influenza A/PR/8/34 virus; all control mice died within five days. As in the previously mentioned study, the HuIFNαB/D did not protect mice that were Mx1^-/-^ [53].

A single intranasal pretreatment with 100 IU of murine IFNα given 8-48 hours before viral challenge reduced lung viral titers and protected BALB/c mice against lethal H5N1 or pandemic H1N1 viral infections. Multiple intranasal pretreatments with murine IFNα enhanced the antiviral effect [54].

Recombinant HuIFNαB/D interferon was given intranasally to guinea pigs at a dose of 500,000 IU/kg body weight. HuIFNαB/D given 36 and 12 hours before, and every other day for seven days after, virus inoculation blocked virus infection in two of four guinea pigs challenged with either H5N1 or 1918 influenza A virus. A single intranasal dose of HuIFNαB/D given 12 hours before H5N1 challenge delayed virus replication 1-3 days, but not 5–7 days post virus inoculation [55].

Ferret IFN given intranasally 20 and 4 h before challenge with 10^5^ TCID_50_ of USSR/77 (H1N1) influenza virus was as good or better than oseltamivir given orally every 12 hours for five days starting four hours prior to virus challenge. At 24 hours, nasal wash virus titers from IFN-treated ferrets were 10 fold lower than washes from oseltamivir-treated ferrets and 100 fold lower than washes from controls. The ferrets given IFN or oseltimivir developed fewer general clinical signs, and the fever peak at day 2 was not observed, compared to controls. IFN-treated ferrets displayed better exercise (running) endurance than oseltamivir-treated ferrets and both IFN and oseltamivir-treated groups had better running times that controls. Additional IFN treatments at 24 and 48 h after virus challenge enhanced the protective effect of IFN. Doses of 10,000,000 IU of HuIFNαB/D were only partially beneficial in ferrets [56]. Lower doses of human or murine IFNα were beneficial in man and animals [38,39,40,41,42,43,44,45,51,52,53]. A better effect in ferrets might have occurred if a lower dose of HuIFNαB/D was given. Pretreatment with ferret IFN at 20 and four hours did not reduce mortality at 6-8 days after virus challenge. Additional IFN treatments after virus challenge may have improved survival; after all, these authors reported that more IFN treatments enhanced the protective effect in ferrets as measured by reduction of nasal wash virus titers [56].

IFNα/β receptor-deficit mice were compared to wild type SvEv129 mice as matched controls. Mice were inoculated with 1000, 100 or 10 MID^50^ of H5N1 viruses A/HongKong/483/97 or A/HongKong/486/97. IFNα/β receptor-deficit mice had a significantly more rapid mean time to death, succumbing to viral infection three days earlier than SvEv127 mice. The authors concluded that the type I IFN response contributed to the early control of H5N1 virus infections, but was insufficient to protect against the fatal outcome in these inbred mice. When virus replication was evaluated at 1, 3, 5, and 7 days post infection in mice given 10 MID^50^ of either virus, the results suggested that type I IFN response controlled the spread and/or extent of viral replication in extrapulmonary organs of H5N1 virus infected mice [57].

Influenza A virus nonstructural protein 1 (NS1) significantly inhibits tripartite motif (TRIM)25 proteins that mediate RIG-1 CARD ubiquitation leading to inhibition of host viral RNA sensor RIG-1. As a result, the NS1 protein enables influenza virus to inhibit host IFN production [58]. Host cell IFNα production is necessary is to upregulate human MxA gene, an IFN-stimulated gene involved in antiviral resistance to influenza viruses. MxA gene expression was not inducible directly by virus but was only inducible by type I or III IFN [59]. Exogenous administration of intranasal or oral IFN may replace host IFN inhibited by NS1 resulting in the benefits reported in man and animals allowing MxA gene to be expressed. 

## 3. Oromucosal IFN Therapy for Respiratory Syncytial Virus and Other Respiratory Tract Infections

The effect of a daily oromucosal dose of HuIFNα2b (10 U/Kg) was tested in a double-blind placebo-controlled study in hospitalized children who had RSV infection. Each patient, in addition to standard therapy, received an oromucosal dose of either HuIFNα or placebo daily for a total of 10 days. On admission each patient was given a score (0-5) according to the severity of symptoms (temperature, respiratory rate, apnea, wheezing, *etc*.). Clinical scores were collected daily during hospitalization and on day 10 of the study. The mean change in score for controls (n = 20) was –0.296 ± 0.333, and for HuIFNα (n = 18) the mean change was –1.198 ± 0.746 (p < 0.0001). The reduction in the severity of disease, as indicated by the scores, was significant in the IFN-treated group, suggesting that children who received HuIFNα recovered more rapidly than the placebo-treated children [60]. One of the authors has continued to treat children with HuIFNα given orally in low doses since 1992 and estimates he has treated over 2,000 children without adverse events [61].

The animal model for studying RSV is the cotton rat *(Sigmodon hispidus)* given the Long strain of human RSV. Cotton rats were given HuIFNα in drinking water before and after challenge with human RSV. Administration of HuIFNα reduced the severity of disease and the amount of recoverable RSV virus in the lung, compared with rats that did not receive IFN. In this study, the lowest dose of HuIFNα evaluated (0.2 U/mL of drinking water) was the most effective [62].

The effect of oromucosal administration of HuIFNα (10 IU/kg body weight) in 22 children (age range 2-14 years) with recurrent acute respiratory tract infections (>6 episodes in previous year) was studied. Duration of HuIFNα therapy ranged from 35–180 days (average 58 days). Treatment with oromucosal HuIFNα was characterized by rapid improvement of clinical and immunological variables. The frequency of respiratory tract infections and duration of illness were also decreased [63].

HuIFNγ has also been used experimentally to treat respiratory virus infections. Preliminary observations showed increased human resistance to respiratory viral infections after treatment of the oropharyngeal cavity with a HuIFNγ solution (about 3 × 10^4^ IU/mL); no other details were provided by the book’s authors [64].

Reports on the oral administration of IFNγ are rare. In a study in mice, a low dose (7 × 10^3^ U/day) of murine (Mu) IFNγ was provided in drinking water to adult HAM/ICR mice starting one day prior to inoculation with *Salmonella* serovar *Typhimurium*. The low dose of MuIFNγ reduced the penetration of Salmonellae into intestinal epithelial cells, development of bacteremia, and mortality rate, and prolonged survival times, compared to control mice [65].

## 4. Oromucosal IFN Therapy in Measles Virus Infection

Thirty (30) confined pediatric patients were prospectively and randomly assigned to either a placebo or IFN group and observed daily for 14 days in a double-blind manner. The IFN patients received a daily oromucosal dose of 200 IU of HuIFNα. The HuIFN-treated group showed shorter average duration of malaise (3.2 *vs*. 10.7 days, p < 0.0001), anorexia (3.1 *vs.* 6.7 days, p < 0.0001), and irritability (1.1 *vs.* 2.2 days, p < 0.01); shorter duration of macular/maculopapular/papular lesions (4.3 *vs.* 8.2 days, p < 0.0001) and branny desquamation (4.6 *vs.* 5.8 days, p < 0.05); and shorter time for rash to become generalized (5.5 *vs.* 10.3 days, p < 0.0001). No hematologic, renal, or liver toxicities were noted. In this study, oromucosal HuIFNα was both safe and effective in children with measles infection [66].

Measles virus can completely suppress the IFNα-induced antiviral state due to suppression of IFNα-inducible gene expression at a transcriptional level. JAK1 phosphorylation induced by IFNα is suppressed in measles virus infected cells [67]. Perhaps administration of exogenous IFNα can partially overcome this viral suppression of host defenses. 

## 5. Oromucosal IFN Therapy in Papillomavirus Infection

Forty patients with acuminata condyloma, a genital mucocutaneous papillomavirus-associated epithelial proliferative disease, were given either placebo or 150 IU HuIFNα as a “mouth wash” which was held in the mouth for 2 minutes tid for 10 days. Six of 11 patients given HuIFNα showed disappearance of “coilocitosis” versus three of 17 in the control group [68]. Cervical human papillomavirus (HPV) infected patients responded to orally administered 150 IU HuIFNα given tid for 60 days significantly better than controls (75% versus 30% “showed colposcopic and histological regression of lesion”) [69]. Cervical HPV infections were treated with laser surgery or orally administered 150 IU HuIFNα, or both. The HuIFNα was held in the mouth for one minute twice daily for 30 days. The complete treatment response to oromucosal HuIFNα was 58.8%, which was comparable to laser surgery (67.2%). When both oromucosal HuIFNα and laser surgery were performed, 72.2% had a complete response [70]. Twenty women were given 150 IU HuIFNα tid as a liquid held in the mouth for 2 minutes, daily for 10 days; 20 other women received placebo. Oromucosal HuIFNα reduced the surface area of cervical HPV lesions and resulted in more complete regression of herpesvirus infections [71]. HuIFNα lozenges (150 IU) given three times daily reduced the number and surface area of papillomas in the oral cavities of HIV-positive patients [72].

The US FDA has approved a natural [73] and a recombinant [74] IFNα to treat acuminata condyloma by intralesional injection. The approved dosage for the natural IFNα is 250,000 IU/lesion twice weekly for up to eight weeks [73]. The approved dosage for the recombinant IFNα is 1 million IU/lesion (in a maximum of five lesions in a single course) three times weekly for three weeks [74]. To achieve results with injectable IFNα, at least 10,000 times more IFN is injected, compared to the reported oral dosage of IFNα.

## 6. Oromucosal IFN Therapy in Human Immunodeficiency Virus (HIV)

Low-dose oromucosal HuIFNα has been tested for effects in 22 clinical trials of HIV+ patients [75,76,77,78,79,80,81,82,83,84,85,86,87,88,89,90,91,92,93,94,95,96]. Weight gain, relief from opportunistic infections and stabilization and/or improvement in blood profiles (CD4+ cell counts, in particular) have been reported as positive benefits of oromucosal HuIFNα therapy in 12 of the 20 studies in which HIV+ symptomatic patients were enrolled and clinical effects were monitored [75,76,77,78,79,83,85,88,90,91,94,96]. In these studies, different sources of HuIFNα and different doses and schedules were tested. In general, African or African-American HIV+ patients [75,76,77,78,79,87,88,90,94] responded more positively to oromucosal administration of HuIFNα than did other HIV+ ethnic groups including Germans [80], Canadians [81,82], Philippinos [86], or Americans [91,93,95]. The reasons for the differences in response to oromucosal HuIFNα when used in Africa versus Europe or North America are unknown but could be related to race, diet and/or concomitant indigenous infections, such as malaria. It is important to note that some human HIV-treatment studies with oromucosal HuIFNα have reported no beneficial effects [80,81,82,86,91,93,95].

## 7. Oromucosal IFN Therapy in Chronic Active Hepatitis

Several publications have reported on the use of HuIFNα lozenges (generally 100-200 IU) in the treatment of hepatitis B patients [97,98,99,100,101,102]. In Poland, generally more than 50% of chronic active hepatitis B patients showed loss of circulating hepatitis B e antigen (HBeAg) and loss of hepatitis B DNA in blood [97,98,99,100,101]. In the Philippines, 400 IU of HuIFNα sublingual tablets given once daily for 8 months, compared to placebo, resulted in significant (p < 0.05) clearance of HBeAg, and development of antibodies against HBeAg [102]. In contrast, one study reported that oromucosal IFNα was not an effective therapy for hepatitis B [103]. The dosage of oral IFNα which achieved seroconversion is at least 10,000 times less than a single injection of IFNα approved by the FDA for the treatment of hepatitis B [104,105]. As reported for acuminata condyloma treatment, the oral route of administration of IFNα achieves similar results at a much lower dosage than that given by injection.

In contrast, attempts to treat hepatitis C virus (HCV) with oral IFNα have been limited and only slight benefits were noted. In five pilot trials, low-dose IFNα given in the form of orally dissolving lozenges was found to be free of significant side effects when given to chronic HCV patients who were treated for up to 19 months. In the two largest studies conducted (15 and 33 patients, respectively), significant decreases in elevated liver enzyme levels were observed overall, or in a sub-group analysis, but sustained normalizations were not observed [106]. In a small study in Poland (n = 6) no changes in aminotransferases were seen, but significant improvement in clinical symptoms was reported [107]. In the remaining two studies, three of 14 (21%) Asian HCV patients had a sustained normalization of aminotransferase levels when treated with 150 IU IFNα lozenges given once daily for up to nine months [106]. In an Australian pilot study on hepatitis C patients, an unusual profile of immediate undesirable side effects was noted and the study was terminated [108]. 

## 8. Oromucosal IFN Therapy in Human Autoimmune Diseases

Lozenges containing 150 IU of HuIFNα given tid have been reported to be beneficial in patients with Sjogren’s syndrome (SS), an autoimmune disease of the exocrine glands [109,110,111,112]. Two double-blind, placebo-controlled clinical trials of HuIFNα lozenges in the treatment of primary Sjögren's syndrome were conducted. Results of both Phase III clinical trials demonstrate an improvement in saliva production in treated patients [109]. A total of 497 patients were treated tid for 24 weeks with a lozenge containing either 150 IU of HuIFNα or a placebo. Analysis of participants who completed the trials, designated as evaluable patients, found a significant increase in unstimulated whole saliva (UWS) production among the HuIFNα treated patients, as compared to those who received placebo. Increases in UWS are important to the Sjögren's patient since UWS represents the basal salivary flow that is present over 90% of the day [112]. Importantly, IFNα treated subjects exhibited a significant correlation between increases in UWS and improvement in a number of the symptoms of Sjögren's syndrome, including oral dryness, throat dryness, nasal dryness and the ability to swallow foods. This finding suggests that patients were able to perceive a benefit of having increased salivary flow [109].

Multiple sclerosis (MS) studies in humans have shown that ingestion of 10^4^ or 3 × 10^4^ IU of HuIFNα decreases Convanavalin A-mediated lymphocyte proliferation and serum ICAM-1 levels. In healthy volunteers, HuIFNα ingested three times per week for two weeks at 10,000 IU/dose resulted in decreased IL-2 secretion and, at 30,000 IU/dose, resulted in decreased IFNγ, TGF-β and IL-10 productions [113]. Ingested HuIFNα decreased MRI brain lesions in MS patients, and a positive treatment effect was noted in MS patients given 10^4^ IU of HuIFNα orally, but not when they were given 3x10^4^ IU [114,115]. MxA MRNA induction and TNF-α mRNA repression was studied in 24 patients with relapsing and remitting MS after they were given 100, 300, 1,000, 3,000 or 10,000 IU of human IFNα by ingestion. The best dose of IFNα for repression of TNF-α mRNA was 100 IU, but the best dose of IFNα for maximum MxA induction was 1000 IU [116]. 

The safety, tolerability and effects on MRI lesions of three different doses of oral IFNβ-la compared with placebo over six months was evaluated in relapsing-remitting (RR) MS patients. In this multicenter, double-blind, randomized trial, RR-MS patients received 0.006, 0.6 or 6 million IU IFNβ-la or placebo every other day for up to six months. Oral IFNβ-la showed neither beneficial effects in RRMS nor any systemic biologic effects [117]. This failure of orally administered IFNβ-la contrasts with the efficacy reported for orally administered HuIFNα in MS patients [114,115]. 

Ingested IFNα at 3x10^4^ IU, given daily or every other day, helped preserve residual beta cell function in Type 1 diabetes patients. Of the 10 newly diagnosed Type 1 diabetes patients, 8 showed significant preservation of residual beta cell function up to 12 months post the initiation of oral IFNα treatment [118]. 

Ingested IFNα given at 5,000 IU daily for a year stabilized B-cell function in children with new onset type 1 diabetes significantly (P < 0.028) better than placebo or 30,000 IU IFNα daily [119]. Adverse events occurred at similar rates in all treatment groups.

## 9. Oromucosal IFN Therapy in Cancer

Cachectic cancer patients have experienced periods of improved appetite and weight gain from liquid HuIFNα given once daily [120]. A crude bovine IFNα/β given tid for four 5-day periods over two months was reported to reduce melanoma in two patients [21]. Even though oromucosal HuIFNα has also been reported to be ineffective in cancer patients [121,122], five of eight patients given low doses of HuIFNα (1-2 IU/kg body weight) experienced an increase in appetite, energy level and general well being, compared to only one of 13 cancer patients given 4-16 IU/kg (P < 0.01) [123].

The effect of oral IFNα was studied in subjects with 5-fluorouracil (5FU)–induced mucositis following 5FU treatment of a solid tumor. Study entry required Grade 2 mucositis to have been observed during the previous course of 5FU. Eligible subjects were given a 150 IU HuIFNα lozenge daily for 14 days beginning on the first day of the next scheduled course of 5FU. Six of 11 subjects were considered positive responders to HuIFNα treatment as they experienced less mucosal damage along with reduced mouth pain during 5FU chemotherapy [124].

## 10. Oromucosal IFN Therapy in Diseases of Unknown Etiology

Patients with fibromyalgia syndrome showed relief in morning stiffness and improved physical function when given daily lozenges of 50 IU HuIFNα [125]. Oromucosal HuIFNα given as a liquid (1,200 IU/day) once daily for 2-6 weeks was an effective therapy for aphthous stomatitis [126,127]. Lozenges containing 150 IU of HuIFNα given daily prevented aphthous stomatitis or gingivitis in HIV+ patients [128]. A cream containing HuIFNα (1,200 IU/mL) was applied twice daily for 4–6 weeks to the oral cavity of five patients with moderate to severe refractory (2-40 years) lichen planus. Relief from lichen planus was noted within a few days and all lesions subsided during a 2-month follow-up interval [129]. These data suggest that IFNα introduced into the oral cavity, besides having systemic effects, may be useful in the treatment of local oral lesions.

## 11. Summary of Oromucosal IFN as a Therapeutic Modality

Many reports have demonstrated that oromucosal or gastric administration of IFN can induce systemic beneficial effects in both animals and humans [22]. It is also clear that additional work is needed to: 1) more clearly delineate the sites and mechanisms of action of oromucosal or gastric IFN, 2) determine optimal doses and schedules, and 3) determine disease indications and circumstances in which beneficial effects can be most reliably achieved.

At present, the best available data suggest that beneficial effects of orally administered IFNα are mediated by local interactions between the administered IFN and certain populations of regulatory cells present in the oropharyngeal mucosa. This IFN-cellular interaction is translated into systemic effects by amplification phenomena secondary to this interaction. Within the oral mucosa, a common intracellular event appears to be induction of 2'5' AS enzyme activity [130,131,132,133,134,135] and upregulation of MHC class I proteins [135] on cells exposed to IFN. Finally, it must be emphasized again that all available data indicate the oromucosal route of administration has significant systemic activity without the troublesome and serious side effects associated with high-dose parenteral therapy. 

An emerging concept is that the positive effects of oral IFN therapy are also critically dependent upon the timing of administration in regards to the stage of the immune or inflammatory stimulus. In general, IFN given prior to encounter with immunogen suppresses immunoglobulin production and class switching by B cells. This is particularly striking in several animal models of asthma [134] wherein IFN pretreatment suppresses the IgE allergen response and inhibits systemic and local eosinophilia characteristic of allergic disease [136]. Similar "protective" effects are seen when IFN is administered to experimental animals prior to challenge with infectious, particularly viral diseases. It is not known if this protective effect is mediated by IFN-enhanced immune responses or by other cytokine-mediated mechanism(s).

In contrast, when IFN is administered during ongoing and misdirected autoimmune and inflammatory diseases of uncertain etiology, IFN-mediated induction of immune suppressor effects are seen wherein suppressor T cells are induced and cytotoxic T cells and the cytokine product of cytotoxic T cells (IFNγ) are reduced. The net effect of this action is to dampen harmful and progressive inflammatory disease and thus to re-establish tissue equilibrium in the affected hosts. This effect is particularly striking in the suppression of relapsing EAE in various animal models [137,138,139,140,141,142,143,144,145] and the suppression of sailoadenitis and lacrimitis characteristic of Sjogren's syndrome in humans [109,110,111,112] and keratoconjunctivitis sicca in dogs [146]. These data suggest that for immune-mediated diseases, the progression of clinical disease can be down-regulated by oral IFNα therapy. 

The antiviral effects of orally administered IFN are also striking and have been demonstrated for both DNA and RNA viruses and in both natural and experimentally induced diseases [22]. It is not clear if the administered IFN exerts its effects directly on virus-infected cells or the more likely case of indirect effects via interactions with the immune system.

Parenteral IFNα is approved by the FDA for treatment of hepatitis B, hepatitis C, genital warts and various cancers [104,105]. The recommended dose of parenteral IFNα for these conditions is typically 3 million IU. Adverse physiologic and psychologic events including suicidal behavior is a significant impediment to wide-spread acceptance by both patients and physicians. In contrast, the lozenge dose of IFNα found to be effective in clinical trials for Sjogren’s syndrome was 150 IU tid daily, approximately 6,700 times less than the amount of IFNα contained in a single parenteral injection dose. In contrast to the experiences with parenterally administered IFNs, oromucosal IFNα in humans has the distinct advantages that it is generally nontoxic and easy-to-administer. In spite of the negative side effects, parenteral administration of cytokines is regarded as a viable therapeutic option for selected human diseases.

**Table 1:** Comparison of Oromucosal and Parenteral Interferon.

Comparison	Oromucosal IFN	Parenteral IFN
Dose	Up to 500 units	Up to 10,000,000 units
Side Effects	Rare/Mild	Common/Severe
Administration	Oral Lozenges	Needle/Syringe
Stability	Stable at room temp	Refrigeration required

For insight into the mechanism of action of orally administered IFN, animal data are helpful. Normal, nude, and SCID mice given recombinant MuIFNβ in their drinking water for three days all had intracellular 2′5′AS activity detected in their liver and whole blood. Normal and sham-operated mice, but not hypophysectomized or adrenalectomized mice, had intracellular 2′5′AS activity detected in their liver and whole blood after administration of recombinant MuIFNβ in the drinking water for 3 days. The authors concluded that the induction of 2′5′AS by oral MuIFNβ was mediated by the hypothalamic-pituitary-adrenal axis in mice [136].

Sixteen to 24 hours after intragastric administration of MuIFNα (10^2^ to 10^4^ units) or ovine IFNτ (10^2^ to 10^5^ units), 2′5′AS was detected in whole blood samples obtained from ICR mice [147]. Other genes are also upregulated after oromucosal administration of IFNα [14,15,16,17,18,19]. For example, the amount of RNA transcripts of the ATP-dependent IFN responsive gene (ADIR) was increased 6-fold in oropharyngeal tissue of Swiss mice four hours after oromucosal administration of MuIFNα (10^5^ units), compared to untreated control mice [14].

Oral administration of IFNα has been shown to change systemic phenotypic expression of lymphocyte populations. IFN-activated natural killer cells, B cells, and T-cell subpopulations are detected in the peripheral circulation of mice with tumors as early as four hours after the initiation of IFNα oromucosal treatment. In addition, oromucosal treatment with IFNα also induced trafficking of cells from the spleen and peripheral lymph nodes to the site of tumor cell replication. Other genes that are upregulated after oral administration of IFNα include genes for chemokines and proteases associated with antigen processing and those involved in lymphocyte activation, apoptosis, and protein degradation [15,148]. Similar observations have also been made in cattle given human IFNα orally [16].

Genes differentially regulated in bovine peripheral blood were identified through the use of cDNA microarrays after oral administration of human IFNα. Thousands of genes were noted to be IFNα regulated. Of these, about 8.5% had a minimum 4-fold degree of change, the majority of which represented novel IFN-stimulated genes (ISG). Several upregulated ISG were transcripts with key and diverse biologic functions, including antigen processing and presentation, leukocyte migration, lymphocyte activation, immune effector and modulation functions, apoptosis, and hematopoiesis. Interestingly, IFNα expression itself was not modulated in bovine peripheral blood, suggesting that the blood levels of IFNα are not the hallmark of the immunostimulatory effects of oral IFNα therapy. Rather, IFNα seems to interact with local mucosal lymphoid cells in the gastrointestinal tract. This interaction may initiate a signaling cascade eventually leading to the transcriptional induction of ISGs, which in turn encode immunostimulatory proteins. Thus, ISGs, through the proteins they encode, may potentially perform critical immune modulation functions [16].

Fish were given HuIFNα at 0.05 IU once daily for three days and blood collected 1, 3, and 5 days after HuIFNα treatment. Administration of a low dose of HuIFNα was found to activate various functions of carp leucocytes including the production of superoxide anion, phagocytosis and phagocytic index. The production of superoxide anion in HuIFNα-treated fish was significantly higher than the control fish at 1 day post-treatment. HuIFNα treatment augmented the expression of cytokine genes in the carp head kidney leucocytes. A significant upregulation of IL-1ß gene expression in the HuIFNα-treated fish on days 1, 3, and 5 post-treatment was observed. TNFα expression was also found to be significantly upregulated in the fish treated with HuIFNα when analyzed on days 1 and 5 post-treatment expression of IL-10 was enhanced in HuIFNα-treated fish when observed on days 1, 3 and 5 post-treatment [18].

## 12. Conclusions

This paper has reviewed the relevant literature on the clinical use of the oromucosal route of administration of IFNs. A better understanding of the applications and potential benefits of this modality are under active investigation. In the particular case of low doses of IFNα, the current influenza pandemics have highlighted the urgency of this work.
